# Classical, Molecular, and Genomic Cytogenetics of the Pig, a Clinical Perspective

**DOI:** 10.3390/ani11051257

**Published:** 2021-04-27

**Authors:** Brendan Donaldson, Daniel A. F. Villagomez, W. Allan King

**Affiliations:** 1 Department of Biomedical Sciences, University of Guelph, Guelph, ON N1G 2W1, Canada; bdonalds@uoguelph.ca; 2 Departamento de Produccion Animal, Universidad de Guadalajara, Zapopan 44100, Mexico; dvilla@cucba.udg.mx; 3 Karyotekk Inc., Box 363 OVC, University of Guelph, Guelph, ON N1G 2W1, Canada

**Keywords:** clinical cytogenetics, genomics, chromosome abnormality, reciprocal translocation, domestic pig

## Abstract

**Simple Summary:**

Chromosome rearrangements are one of the main etiological factors leading to impaired fertility in the domestic pig. The high prevalence of chromosome rearrangements in swine herds, coupled with the production of significantly lower litter sizes, has led to the implementation of cytogenetics techniques in screening prospective breeding boars for rearrangements. Beginning in the 1960s, classical cytogenetics techniques have been applied in laboratories, resulting in the identification of over 200 distinct chromosome rearrangements in the pig. More recently advances in technology, and the development of molecular cytogenetics and cytogenomics techniques, have enhanced the resolution of rearrangements and advanced diagnostic techniques, allowing for more precise and rapid diagnosis of rearrangements.

**Abstract:**

The chromosomes of the domestic pig (*Sus scrofa domesticus*) are known to be prone to reciprocal chromosome translocations and other balanced chromosome rearrangements with concomitant fertility impairment of carriers. In response to the remarkable prevalence of chromosome rearrangements in swine herds, clinical cytogenetics laboratories have been established in several countries in order to screen young boars for chromosome rearrangements prior to service. At present, clinical cytogenetics laboratories typically apply classical cytogenetics techniques such as giemsa-trypsin (GTG)-banding to produce high-quality karyotypes and reveal large-scale chromosome ectopic exchanges. Further refinements to clinical cytogenetics practices have led to the implementation of molecular cytogenetics techniques such as fluorescent in-situ hybridization (FISH), allowing for rearrangements to be visualized and breakpoints refined using fluorescently labelled painting probes. The next-generation of clinical cytogenetics include the implementation of DNA microarrays, and next-generation sequencing (NGS) technologies such as DNA sequencing to better explore tentative genome architecture changes. The implementation of these cytogenomics techniques allow the genomes of rearrangement carriers to be deciphered at the highest resolution, allowing rearrangements to be detected; breakpoints to be delineated; and, most importantly, potential gene implications of those chromosome rearrangements to be interrogated. Clinical cytogenetics has become an integral tool in the livestock industry, identifying rearrangements and allowing breeders to make informed breeding decisions.

## 1. Introduction

The domestic pig (*Sus scrofa domesticus*) is known to have a high proportion of chromosomal rearrangements relative to other species [1]. Chromosome rearrangements are structural chromosome abnormalities that result from the breakage of one or more chromatids and a subsequent rearrangement or ectopic exchange of chromosome segments. This results in the production of derivative chromosomes (e.g., translocation chromosomes), a gross change in the karyotype of the carrier that is often visible under light microscopy. Despite the large-scale rearrangement of genetic material within the genome, often on the scale of millions of base pairs, chromosome rearrangements typically occur with no associated observable signs of their presence [2]. Nevertheless, there are some reports describing chromosome abnormalities associated with physical malformations [3].

Most of the time, chromosome rearrangements appear to occur with minimal losses of genetic material, and thus produce no physical malformations typically associated with aneuploidy. Chromosome rearrangements are, however, known to cause predictable fertility loss in carriers. The litter size losses caused by chromosome rearrangements are variable among carriers and are dependent on a variety of factors, including the morphology of the rearrangement [4]. The derivative chromosomes must satisfy the need for homologous chromosomes to pair during meiosis and do so with a variety of complex formations between derivative chromosomes and their counterparts [5]. Asymmetric segregation of these chromosomes during meiosis leads to a subset of gametes being genetically unbalanced [6]. Resulting unbalanced embryos, or those with lethal mutation due to rearrangement, die early during post-fertilization development due to the presence of genetic imbalances—partial aneuploidies—from derivative chromosomes. Chromosome rearrangements, most notably reciprocal translocations (i.e., balanced exchanges between non-homologous chromosomes), are one of the leading causes of reproductive dysfunction in the domestic pig, with 50% of hypoprolific boars estimated to be carriers [2]. Balanced reciprocal translocations are known to cause the largest litter size losses, averaging 40% piglet loss relative to the herd average, while other chromosome rearrangements have a lesser yet significant impact on litter size [4,7].

Chromosome rearrangements are known to occur in various mammalian species, with reciprocal translocations being especially prevalent in the domestic pig relative to other species. Chromosome rearrangements, including balanced reciprocal translocations, are expected to occur frequently in swine herds throughout the world, being proposed to occur spontaneously in 1/200 live births [8,9]. Carriers of rearrangements, if permitted to breed, may then pass on the rearrangement to approximately 50% of their successful offspring, increasing the prevalence of chromosome rearrangements in swine herds over time [10]. In order to reduce the presence of chromosome rearrangements in swine herds, several labs operate cytogenetic screening programs in order to screen prospective breeding boars for chromosome rearrangements [11]. Although screening programs cannot totally eliminate rearrangements from herds, they can prevent carriers from breeding, resulting in the maintenance of litter size, eliminating the possibility of inheritance, and reducing the prevalence of rearrangements. In countries where such programs are available, many breeders will voluntarily submit their breeding boars for cytogenetic screening, seeing clear economic benefits to managing the presence of rearrangements [7]. Most large swine producing countries, however, fail to implement cytogenetic screening of their swine herds. Thus, the implementation of cytogenetic screening, or some other methods, to identify carriers or potential carriers have room to be widely implemented in the swine industry and greatly reduce the impact of chromosome rearrangements on swine herds.

The field of clinical cytogenetics seeks to apply various laboratory, molecular, and genomic techniques to the study of chromosome rearrangements in order to understand their possible implications on gene and genome functionality. Clinical cytogenetics laboratories, though relatively sparse and underutilized, serve an important purpose to identify chromosomal rearrangements and other chromosome abnormalities in the pig genome, to not only assist selection of boars for breeding services but also to help further the study and understanding of chromosome rearrangements in the domestic pig, including their breadth, diagnosis, origins, and the effect they have on meiosis and the genome itself.

## 2. Classical Cytogenetics

Conventional laboratory techniques applied to effectively view chromosomes under a light microscope are referred to as classical cytogenetics techniques. This encompasses the in vitro culture of cells such as peripheral blood lymphocytes and fibroblasts, and the subsequent preparation of chromosomes using a series of staining and banding techniques allowing metaphase chromosomes to be effectively viewed under a common light microscope. The first cytogenetics techniques employed in the pig used fixed testicular material and non-differential stains such as crystal violet, allowing the chromosomes to be differentiated from their surroundings, enabling descriptions of the general morphology and the determination of the diploid chromosome number [12]. The diploid chromosome number of the pig is 2*n* = 38, consisting of 18 autosomal chromosome pairs, which vary in length and morphology (12-bi-armed and 6 one-armed pairs), and two sex chromosomes, XX or XY [12,13,14]. The employment of classical cytogenetics techniques to properly determine the diploid chromosome number in mammalian species, especially humans, was essential for the later development of clinical cytogenetics, which linked chromosomal aneuploidy such as trisomy of human chromosomes 13, 18, and 21 to known diseases and later linked sex chromosome aneuploidy and chromosome rearrangement to infertility [15,16,17,18,19,20].

Following the development of modern in vitro cell culture techniques, providing high-quality, well-spread metaphase chromosomes on glass slides [21,22], researchers began to employ banding techniques and differential staining in order to distinguish chromosomes from one another and observe those chromosomes at a higher resolution. One of the first banding techniques introduced was the hybridization of quinacrine mustard (QM) to chromosomes, which produced a distinct fluorescent pattern on each chromosome as a function of the relative density of guanine residues across each chromosome [23]. The resulting technique was referred to as quinacrine fluorescence (QFQ), or Q-banding. Soon after, other differential banding techniques were developed, including Giemsa-trypsin banding (GTG), or G-banding [24]; replication banding with Giemsa staining (RBG), or R-banding [25]; and reverse-banding with acridine orange staining (RBA), or R-banding [26].

GTG-banding technique employs the proteolytic enzyme trypsin to partially digest the chromosomes, and Giemsa stain, producing a distinct banding pattern on each chromosome where the condensed heterochromatic regions of chromosomes characterized as less transcriptionally active, late-replicating, and AT-rich are stained more intensely than the less condensed euchromatic regions characterized as more transcriptionally active, early-replicating, and GC-rich [24,27,28]. RBA-banding employs the use of the protease trypsin similar to GTG, and acridine orange fluorochrome to stain euchromatic regions more intensely, resulting in a banding pattern that is the inverse of GTG-banding [25,26,28]. RBG-banding employs bromodeoxyuridine (BrdU), which incorporates into DNA, substituting for thymidine residues, and Giemsa to stain regions of the chromosome where BrdU has incorporated, staining AT-rich heterochromatin deeply, producing R-bands [26]. Although these banding patterns were initially developed for the examination of human chromosomes, these methods have been adapted for use on porcine chromosomes and are still routinely applied to the study of porcine chromosomes [5,29].

Chromosomal banding techniques may also be complemented with a variety of staining protocols selective for specific chromosomal regions [30]. These staining techniques may be used to reveal chromosomal features such as constitutive heterochromatin blocks through the use of barium hydroxide (C-bands, CBG technique), nucleolar organizing regions by employing silver-staining techniques (Ag-NOR-bands, Ag-I technique), and telomeric regions through thermal denaturation (T-bands, THA technique) [31,32,33].

RBA-banding and especially GTG-banding are the most common banding techniques employed in porcine conventional cytogenetics. These banding methods produce approximately 300 bands across all chromosomes, thus the resolution provided by these methods is referred to as the 300 band-level [34]. More specifically, standard GTG-banding and RBA-banding produce a resolution of approximately 5–10 Mb, with chromosome features or rearrangements under 5 Mb in size typically t being indistinguishable [28,35]. Less condensed pro-metaphase chromosomes obtained through the use of replication or condensation blockers preventing cells from progressing to metaphase may be used to provide a higher resolution look at the structure and organization of chromosomes [36]. Pro-metaphase chromosomes may be banded using the GTG or RBA banding methods, producing more finely banded chromosomes with 600 total bands, increasing the resolution to 2–5 Mb [28,37,38]. These higher resolution banding methods enable more refined chromosome analysis in order to more accurately determine the sites of chromosome breakage and establish gene loci.

Classical cytogenetics banding techniques have been used to produce standard karyotypes of the pig, as the banding patterns allow for homologous chromosomes to be paired and their banded patterns converted into standard ideograms [30]. The first standard karyotype arranged for the pig used the QFQ-banding technique popular in the early years of porcine cytogenetics [39]. Previous to this, chromosomes were often arranged in an order that varied among laboratories and often simply ordered the chromosomes by length. Additional karyotypes were arranged using novel banding methods, including GTG-banding [30] and RBA-banding [40,41]. The standard application of the GTG and RBA banding techniques to porcine chromosomes, in tandem with the guidelines provided by the Reading conference [42], resulted in the establishment of a standard karyotype of the domestic pig and schematic representations of GTG and RBA-banded chromosomes [34]. Along with these standard karyotypes was the development of one of the first banding nomenclature systems in domestic species [34]. The development of a distinct nomenclature system allowed porcine cytogeneticists to characterize and report chromosome rearrangements and aberrations in a standardized way easily reported to and understood by other cytogeneticists, helping to further develop the field of clinical cytogenetics of the pig.

## 3. Chromosome Rearrangements in the Domestic Pig

Chromosome abnormalities, particularly structural chromosome rearrangements, are remarkably prevalent in the domestic pig relative to other species, with over 200 distinct structural rearrangements in the pig genome being identified [1,43]. The prevalence of structural chromosome rearrangements is variable between countries ranging from 0.47% to 3.3% and is largely influenced by access to screening laboratories that identify carriers and removal affected boars from breeding eligibility [8,11,44]. A variety of chromosome rearrangements are known to occur in the pig, including reciprocal translocations, Robertsonian translocations, tandem fusions, inversions, and deletions of chromosomes [1]. The tendency for pigs to experience balanced reciprocal translocations at the largest frequency results in a large variety of rearrangements reported, with all chromosomes of the pig known to be susceptible to ectopic rearrangements [45].

## 4. Clinical Cytogenetics

The recognized association between chromosome rearrangements and lower fertility has led to the development of routine cytogenetic screening programs in several countries [11]. Worldwide, cytogenetics laboratories primarily apply conventional cytogenetics techniques such as GTG-banding to screen young boars for chromosome rearrangements prior to entering artificial insemination (AI) centres. The National Sow Herd Management Program in France was the first of such cytogenetic screening programs and mandated that boars siring litters of eight piglets or less on average are to be cytogenetically examined prior to servicing additional sows [46,47]. This program has greatly increased the number of boars subject to cytogenetic examination in France over the years and resulted in 20 reported reciprocal translocations in French boars by 1999 [10,48]. This program was expanded in 1999 to include mandatory cytogenetic screening of all boars born of small litters prior to approval for A.I [7]. The success of this program is shown by the reduction in the prevalence of chromosome rearrangements in French boars, with France reporting the lowest prevalence of rearrangements, 0.47%, amongst countries reporting cytogenetic screening results [8]. Many French breeders now voluntarily submit boars for cytogenetic investigation regardless of whether the criteria for screening is met [7,49].

Clinical cytogenetics laboratories have been established in many countries, and at least seventeen countries have laboratories reporting rearrangements in the domestic pig. The countries with the largest cytogenetic screening programs include France, Poland, the Netherlands, Canada, and Spain, with several other countries, including Finland and the United Kingdom, also screening boars for rearrangements. As of 2017, the largest cytogenetic screening program for pigs is conducted at the National Veterinary School of France in Toulouse, with 31,000 boars having passed through this lab as of 2017 [50]. Other large cytogenetic screening programs take place in Spain, the Netherlands, Poland, and Canada, screening 800, 1000, 2000, and 7000 boars, respectively [4,11,43,44]. Clinical cytogenetics programs, although time-consuming, are effective at reducing the prevalence of rearrangements in herds and are cost-effective for breeders, with a cost-benefit ratio of 5.3/1 due to reduced losses from breeding carriers [9]. As of 2021, there are a limited number of cytogenetics laboratories that report the effects of pig screening programs on the prevalence of rearrangement within herds and describe individual chromosome rearrangements [51]. Despite these labs performing cytogenetic screening for breeding boars, just a small percentage of breeding boars worldwide are subject to cytogenetic screening. Further advances in technologies applied in cytogenetics laboratories that reduce the cost of screening, or allow for more rapid results, alongside awareness of the efficacy of cytogenetic screening may increase adoption amongst large swine breeders.

## 5. Reciprocal Translocations

**Figure 1:**
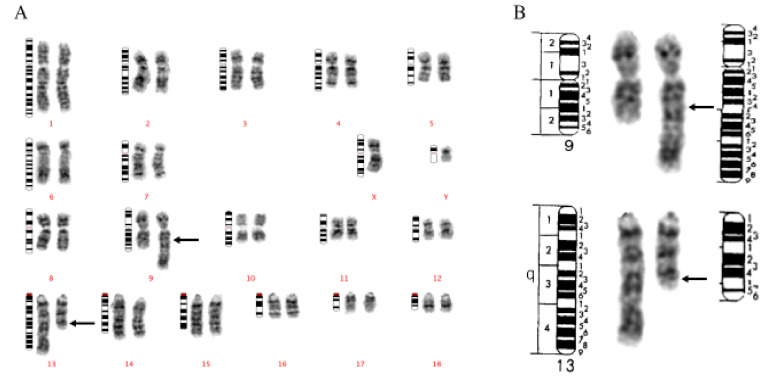
(**A**). GTG-banded karyotype of a Duroc boar carrying a t(9;13)(q24;q31). Derivative chromosomes are placed to the right. Arrows indicate presumptive breakpoints. (**B**). GTG-banded chromosomes of chromosomes 9, 13, and the derivative chromosomes formed by the reciprocal translocation event. The ideogram to the left of each chromosome pair indicates the normal chromosome structure. The ideogram to the right of each chromosome pair indicates the derivative chromosome structure. Arrows indicate presumptive breakpoints. Chromosome ideograms adapted from ref. [34].

Reciprocal translocations are the most prevalent chromosome rearrangement known to occur in the pig, representing 84% of all observed structural rearrangements [8]. Reciprocal translocations result from an exchange of chromatid segments between two non-homologous chromosomes following simultaneous chromatid breaks producing two novel derivative chromosomes (Figure 1). All chromosomes of the pig are susceptible to reciprocal translocation, although chromosomes 7, 10, 12, 14, and 17 appear more susceptible to rearrangement than chromosomes 2, 8, 9, 18, and Y [43,45]. Reciprocal translocations typically have unique breakpoints with just one rearrangement, t(12;14)(q13;q21), being observed in two unrelated boars [8,43].

Balanced structural chromosome rearrangements are a leading cause of fertility losses in pigs, particularly reciprocal translocations, with carriers experiencing average litter size losses of 40% (ranging between 10–100%) relative to the herd average [7,43]. In the case of reciprocal translocations, during meiosis the normal and derivative chromosomes form a quadrivalent shape as a result of full homologous pairing, which progressively may be segregated in a variety of ways, including alternate, adjacent-I, adjacent-II, 3:1 or 4:0, which allow for a high proportion (approximately 50%) of unbalanced gametes to be formed. The exact litter size losses are difficult to predict, with the morphology of chromosomes, size of the rearranged fragments, and involvement of the Y chromosome known to result in complete infertility, proposed to influence piglet loss [52]. The significant loss of litter size and high prevalence of reciprocal translocation in swine herds is one of the principle reasons for the adoption of routine cytogenetic screening programs for young boars prior to entering A.I centres [11].

## 6. Robertsonian Translocations and Tandem Fusions

Robertsonian translocations are a ubiquitous feature of the wild boar, known to have diploid chromosome number of 2*n* = 36, or 2*n* = 37 in the case hybridization with domestic hybrids, primarily due to the presence of rob(13;17) wild boar rearrangement in the homozygotic or heterozygotic state, respectively [53,54]. Robertsonian translocations, and especially tandem fusions, are by contrast relatively rare chromosomal events in the domestic pig. Just seven cases of Robertsonian translocation have been described in the pig, primarily cases of the same rob(13;17) rearrangement endemic in wild pigs [4,8,55,56,57]. Robertsonian translocation is known to occur in the acrocentric chromosomes 13, 14, 15, 16, 17, and 18. In these cases, the chromosomes fuse at the centromeric region, resulting in the production of two derivative chromosomes, a large bi-armed chromosome, and a secondary short chromosome often lost in subsequent cell divisions. Thus, the karyotype of a domestic pig carrying Robertsonian translocation has a distinct 2*n* = 37 diploid chromosome number, with the noticeable addition of a novel large bi-armed chromosome. Tandem fusion occurs similarly to Robertsonian translocations; however, it instead involves the fusion of the telomeric region of one chromosome to the centromeric region of another. Tandem fusion is a rare event in the pig, with just one such rearrangement reported, 37, XY, der(14;17)(q29;q10) [58].

As with other chromosome rearrangements, carriers of Robertsonian rearrangements experience subfertility; however, the effect is less severe than in carriers of reciprocal translocations [4,57]. The trivalent formed by the Robertsonian rearrangement during meiosis segregates asymmetrically but results in a smaller percentage of unbalanced gametes that is sex-dependent, with males having a lower proportion of unbalanced gametes [3.2%] relative to female carriers [28.9%] [59]. The higher proportion of balanced gametes in Robertsonian translocations results in less severe litter size losses (5–22%) relative to carriers of reciprocal translocations [2,4,60,61].

## 7. Paracentric and Pericentric Inversions

Cases of chromosome inversion are relatively rare in the domestic pig with only 12 cases reported in the pig [8]. Chromosomal inversions may be divided into pericentric, involving the centromere, and paracentric, not involving the centromere. In cases of inversion there is no exchange of chromatid segments. Instead, inversions occur as a result of a broken chromatid segment that rotates 180 degrees and reattaches to the original chromosome. Carriers of chromosomal inversions experience minimum litter size loss amongst chromosome rearrangement carriers. Although inversions may result in a proportion of unbalanced gametes during meiosis, the overall proportion is estimated to be small, with average litter size losses of approximately 4% relative to the herd average [2,4,60,61].

## 8. Chromosomal Aneuploidy

Numerical chromosome aneuploidy in the pig is relatively rare and largely confined to observations in embryos and, in the case of live-birth, the sex chromosomes [62,63,64,65,66]. Cases of whole chromosome aneuploidy 37,X, X-chromosome monosomy [63], and 39,XXY Klinefelter Syndrome have been reported [64,65,66]. Cases of aneuploidy involving autosomal chromosomes are rare in the pig, as even partial chromosome aneuploidy, such is the case in the embryos of rearrangement carriers, is not tolerated by the pig. A handful of cases of live boars carrying a partial autosomal aneuploidy have been observed in the pig, the result of inheritance of an unbalanced rearrangement involving short segments in the telomeric regions [3,67,68]. In these cases, aneuploidy is accompanied by physical malformation such as cleft palate [3,68].

## 9. Mosaicism

Chromosome rearrangements are also known to occur in somatic cell lines. Mosaic chromosome rearrangements are estimated to occur frequently in pigs, with limited screening of karyotypes revealing mosaic rearrangements in the karyotypes of 1/300 boars [69]. Mosaic rearrangements arise in somatic cells, rather than the germ line, often appearing confined to certain cell types, such as peripheral blood leukocytes, and thus are not heritable. Indeed, carrier pigs experimentally bred were shown to have offspring with a normal karyotype composition [69]. In addition, mosaic rearrangements have a tendency for recurrence, with three mosaic rearrangements, t(7;9); t(7;18), and t(9;18), being shown to occur recurrently in swine herds [69,70]. Mosaic rearrangements share the same tendency for constitutional rearrangements to experience reciprocal translocation at the highest rate, with no current cases of Robertsonian translocation or inversion being observed in somatic cells. Mosaic rearrangements interestingly appear to tolerate conformations not seen in constitutional rearrangements, including a case of rearrangement between homologous chromosomes, t(7;7) [69]. Mosaic aneuploidies of the sex chromosomes have also been observed in a handful of cases [65,71]. Chimerism, the presence of two distinct sets of DNA in blood leukocytes, has also been described in the form of XX/XY individuals [8,69,72,73,74,75,76]. Somatic or mosaic rearrangements are well known in humans, and are often associated with cancers, especially leukemias and lymphomas that result from the aberrant rearrangement of genes [77]. Although there is no concrete evidence of a relationship between mosaic rearrangements and cancer in the pig, recurrent somatic rearrangements described above are homologous for recurrent somatic rearrangements associated with leukemias in humans [69,70,78].

## 10. Fragile Sites

Fragile sites are heritable chromosome regions known to break under exposure to distinct chemical stressors such as aphidicolin, bromodeoxyuridine (BrdU), and folate [79,80,81]. Sixty of these fragile sites are considered common amongst pigs and are expected to occur in most individuals [81]. Analysis of fragile sites in pigs has shown that cytogenetic bands harboring common fragile sites often overlap with known reciprocal translocation breakpoints, and the presence of fragile sites may be associated with higher frequency of rearrangement in those chromosome regions [4,43,81]. As such, it has been suggested that a subset of chromosome rearrangements in the pig are the result of the breakage of fragile chromosome regions as a response to chemical toxins present in farm environments [4,79,80,82].

## 11. Molecular Cytogenetics

Classical cytogenetics techniques have been instrumental in the development of clinical cytogenetics in the pig and the identification of structural chromosomes rearrangements; however, the limited resolution (>5 Mb) provided by classical cytogenetics impaired the ability to resolve rearrangements at the highest level. Molecular cytogenetics techniques developed in the 1980s were initially applied for physical gene mapping to chromosomes and were later implemented in clinical cytogenetics for the detection of chromosome rearrangements [83]. Molecular cytogenetics as a whole operates around two principles: the target and the probe. Molecular probes are developed to target regions as large as a whole chromosome, or more specific chromosome regions such as the centromere or a specific gene locus [84]. Probes are fluorescently labelled directly with fluorochromes or indirectly with molecules that bind to the probe via fluorochrome-conjugated antibodies [85]. The specificity of the probe for the target is based around the principle of DNA complementary base-pairing, whereby the nucleic acids of fluorescently labelled probes hybridize to the complementary DNA of the target, producing a specific fluorescent signal on the chromosome regions bound by the probe. The specificity of probes for targets enables molecular cytogenetics to achieve a much higher resolution (0.5–10 Mb) than can be obtained through the classical banding techniques [5,28,83].

**Figure 2:**
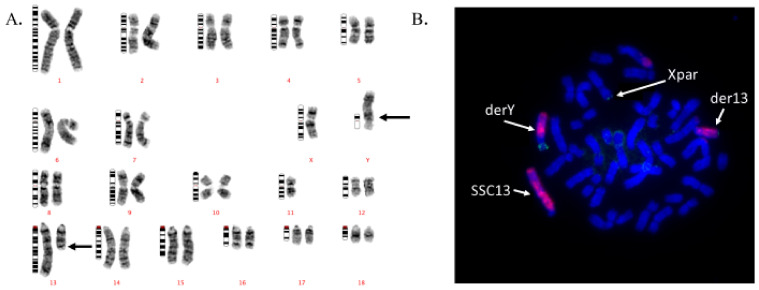
(**A**). GTG-banded karyotype of the t(Y:13) translocation carrier boar. (**B**). FISH chromosome painting of a metaphase plate from the t(Y:13) carrier, green signal pains the Y-chromosome segments, and redish signal pains the SSC13 chromosome segments. The presumptive psuedoautosomal region (green dots) on the X-chromosome is depicted.

In the pig, the most common technique for molecular cytogenetics analysis is fluorescent in situ hybridization (FISH) and primed in-situ labelling (PRINS) [5]. The DNA-probes used for FISH include whole chromosome painting probes and probes obtained by cloning genomic DNA inserts from genomic libraries. The probes obtained from genomic libraries may vary in size depending on the origin and the purpose, and may include cosmid probes with DNA insert sizes of <20–40 kb, bacterial probes, or bacterial artificial chromosome (BAC), with DNA insert sizes of 100–300 kb [86]. Chromosome-specific painting probes may be obtained through flow sorting chromosomes, a process that applies dyes to metaphase chromosome suspensions then runs the suspension through a flow cytometer with a laser exciting the chromosomes, and sorting the chromosomes according to relative amounts of genetic material present, roughly corresponding to the length of chromosomes [87,88,89]. Other techniques for the generation of chromosome painting probes include needle microdissection, which dissects a whole chromosome out of the nucleus or part of a chromosome such as an arm or band using a glass needle [90,91,92], and laser microdissection, which uses a laser to cut out a chromosome or chromosome arm from the metaphase cell [93,94]. Chromosome painting probes for one, two, or the whole chromosome set may then be applied to metaphase spreads. Different colored fluorescent probes may be applied such as single-color (one chromosome), dual-color (two chromosomes), or multi-color (three or more chromosomes) in order to visualize chromosome rearrangement within the genome [95] (Figure 2).

PRINS is a technique that anneals short unlabeled oligonucleotide probes to complementary DNA sequences, which are subsequently extended by Taq DNA polymerase [96]. The PRINS technique is most useful for identifying repetitive DNA sequences such as telomeric and centromeric sequences [97,98]. In the pig, oligonucleotide probes for use of the PRINS technique are available, for telomeric (TTAGG)n repeats, centromeric sequences, and a subset of autosomal chromosomes (1, 9, 11, 14) and sex (Y) chromosomes [83,98,99,100,101]. Most often, PRINS is used as an alternative technique to FISH, for similar applications in the observation of chromosome rearrangements, and gene loci, with the focus on rearrangements located near repetitive sequences in the genome [102].

Another molecular cytogenetics technique that has been applied in the pig is interspecies in-situ hybridization (Zoo-FISH), which applies human genomic probes that hybridize to homologous sequences in animal genomes [103,104]. The use of Zoo-FISH has been used to study the evolution of mammalian karyotypes by analyzing regions of chromosome synteny between species, and for identifying chromosome segments shared by a common ancestor [104]. However, the expanded availability of flow sorted and microdissected chromosome probes available that are specific for the pig have resulted in Zoo-FISH being rarely implemented in the pig [89]. Another molecular cytogenetics technique, sperm-FISH, applies fluorescently labelled probes to decondensed sperm heads, allowing for the analysis of the sperm chromosome constitution [105]. The FISH technique may also be applied to the chromosomes of oocytes and embryos to analyze chromosome composition.

## 12. Implementation in Clinical Cytogenetics

Molecular cytogenetics techniques such as FISH have become essential diagnostic tools for the study of pig chromosome rearrangements, allowing for high-resolution viewing of chromosome rearrangements, their meiotic products, and the more accurate diagnosis of rearrangement breaks (e.g., delineation of chromosomal structural changes) [83]. Molecular cytogenetics is typically performed in a manner complementary to that of the classical cytogenetics techniques, and is most often applied in order to refine and/or verify the breakpoints of chromosome rearrangements originally discovered using banding techniques. The use of chromosome painting probes for FISH, or probes for centromeric and telomeric sequences for PRINS, have been used to examine and refine over 20 chromosome rearrangements [5]. The first instance of FISH being used in this way to study porcine rearrangements was by Konfortova et al. [106], who utilized single-colored painting probes in order to visualize the reciprocal exchange of a t(7;15)(q24;p12). Additional experiments applied flow-sorted probes for dual-color chromosome painting to demonstrate the exchange of small terminal chromosome segments not clearly visible via the classical banding techniques, and to verify several reciprocal translocations originally identified through GTG and RBA banding [7,10,48,49,107]. In these cases, the use of FISH was able to detect small exchanges of chromosome material and pinpoint breakpoints with greater accuracy than is available using banding techniques alone. Additionally, flow sorted dual color probes were used in this instance to correctly identify the breakpoints of a rearrangement, originally delineated as t(11;16)(p14;q14), to t(11;16)(p12;q12) [108]. The use of probes for centromeric sequences and the PRINS technique has also been incorporated into cytogenetic analysis to identify breakpoints and subsequent repositioning of the centromere in a rearrangement involving two pig chromosomes [108].

Other instances of cytomolecular analysis using DNA-probes generated by laser microdissection have been used in the analysis of mosaic rearrangements in pigs. Here, laser-microdissected probes specific to chromosomes 7, 9, and 18 were employed to identify three mosaic rearrangements, t(7;9), t(7;18), and t(9;18), amongst thousands of metaphase spreads [77]. Microdissected probes have also been used for the PRINS technique, with telomeric probes labelling the (TTAGG)n telomeric repeat sequence used to confirm the diagnosis of a reciprocal translocation previously identified through banding techniques t(7;13)(q13;q46) [102]. Notably, inversions may be difficult to discern in a banded karyotype due to there being no inter-chromosomal exchange, making it harder to compare chromosome banding patterns. The use of two painting probes, each specific to a chromosome arm obtained through glass-needle microdissection, have been employed to verify the presence of a peri-centric inversion inv(4)(p14;q23) [92]. Another instance of an inversion being re-examined using cytomolecular techniques was a paracentric inversion that employed BAC probes corresponding to microsatellite markers [109].

Although the application of molecular cytogenetics techniques to porcine chromosomes is typically done complimentary to GTG-banding, recent developments have been made to produce a FISH screening assay of multiple BAC probes specific for the subtelomeric regions of each chromosome arm to rapidly identify any chromosome rearrangement without the need to arrange karyotypes [110]. Preliminary research has shown that this method is useful for diagnosing reciprocal chromosomal rearrangements, and may enable the accurate detection of sub-microscopic rearrangements involving small telomeric exchanges of chromosome material near impossible to detect using classical banding techniques. The application of this assay confirmed the presence of four rearrangements originally identified through GTG banding while identifying a fifth rearrangement, involving small telomeric regions of pig chromosomes 5 and 6 not originally detected through GTG banding [110]. New research into the application of molecular cytogenetics into clinical cytogenetic screening laboratories may thus enable the rapid identification of chromosome rearrangements, helping to reduce the time and labor necessary for the production of GTG-banded karyotypes of each animal.

FISH may also be employed to analyze the interaction of chromosome rearrangements in germ cells, through analysis of synaptonemal complexes and meiotic segregation patterns in spermatocytes. Analysis of the meiotic segregation patterns of chromosome rearrangements provides an estimate of the prevalence of unbalanced gametes, facilitating estimates of fertility loss in carriers [111,112]. The application of FISH to synaptonemal complexes has revealed a complete loss of fertility in three carriers of reciprocal translocations involving the Y-chromosome, t(Y;1) [111], t(Y;14)(q11;q11) [113], and t(Y;13)(p13;q33) [52], and has been applied to other reciprocal translocations such as a t(3;15)(q27;q13) and t(12;14)(q13;q21), revealing expected losses of fertility of 47.83% and 24.33% relative to herd averages, respectively [114]. This technique has also been applied to non-reciprocal rearrangements such as a Robertsonian translocation rob(13;17) and paracentric inversions such as inv(2)(q13;q25), revealing less significant fertility losses of 2.96–3.83% and 4.12% respectively [59,115,116]. These studies have been essential in quantifying expected losses of fertility characteristic of each rearrangement, including analysis of the meiotic segregation profiles of male and female carriers, and comparing fertility losses between types of rearrangements and between the sexes [59,107,116].

Zoo-FISH is rarely used in porcine cytogenetic analyses except in rare instances such as to confirm the diagnosis of a tandem fusion-translocation der(14;17)(q29;q10) previously identified using GTG-banding [117]. A similar approach was used for Robertsonian translocation 15;17 in a European wild boar with a karyotype 37,XY,rob(15;17) in which human painting probes for the homologous chromosomes in the pig were used to demonstrate the fusion of the chromosomes and complement the initial diagnosis based upon GTG-banding [118]. In pigs, sperm-FISH has been used to validate the purity of flow cytometrically sorted boar sperm [119,120], to estimate the rate of aneuploidies in normal individuals [121], and to analyze meiotic segregation patterns in translocation and inversion carriers [114,115,122,123].

## 13. Cytogenomics

In recent years, cytogenomics, which refers to the use of DNA microarrays and whole genome sequencing (WGS) tools to visualize the genome at a high resolution, has increased in prominence. Cytogenomics tools allow the genome and structural variants within to be visualized at a higher resolution than molecular cytogenetics techniques, with resolutions of 100 kb in case of DNA microarrays and nucleotide level resolution in case of WGS [124,125]. The development of a high-quality annotated reference pig genome built upon the foundation laid by the development of autosomal radiation hybrid, recombination, cytogenetic, and BAC maps has been key to the implementation of cytogenomics technologies [126,127,128,129,130,131]. Genome sequences of the pig have been integral to the development and implementation of single nucleotide polymorphism (SNP) chips, and the WGS efforts in the pig providing a framework for genetic and structural variant discovery, and linkage with gene function, have helped to unravel the genetic and genomic factors associated with complex and disease traits [132,133].

DNA microarrays are tools used to analyze genomes consisting of a series of DNA probes attached to a solid surface (chip). SNP-arrays such as Illumina SNP-array genotyping use single-stranded DNA hybridized to fluorescently labelled DNA probes, producing a fluorescent signal that can be read and interpreted, providing an indication of the relative amount of genetic material present corresponding to a nucleotide base at each probe [134]. SNP array genotyping is most often employed to perform association studies between SNP genotypes and disease traits; however, it may also identify copy number variants (CNV), deletions, and duplications of genetic material, and unbalanced rearrangements (partial monosomy or trisomy) at probes expressing higher or lower signal intensity corresponding to proportional changes in the amount of genetic material. Genomic selection, which analyzes associations between tens of thousands of SNPs and specific trait variations in a phenotyped population, is one of the most widely adopted applications of SNP array genotyping in pigs. Genomic selection incorporates phenotypic and genotypic data from pigs and applies regression analysis to estimate the effect of a genotype on a phenotype, resulting in the estimated breeding value (EBV) used to select candidate breeding animals, resulting in genomics-enabled genetic improvement [135,136,137]. Initially introduced for application in dairy cattle breeding, genomic selection is now being used in many sectors within animal and plant breeding, including leading pig breeding companies [138,139]. Genomic selection in the pig has been continually improved through novel genome-wide association studies analyzing novel and refining established phenotype-genotype associations in the pig. These studies primarily focus on economically important traits such as back-fat thickness [140] and meat quality [141], and reproductive traits such as farrowing interval [142].

Comparative genomic hybridization (CGH) similarly uses competitive hybridization between normal and cancer cells to fluorescently labelled probes. The ratio of red-green fluorescence along a chromosome would then indicate the presence of gains or losses of genetic material in the chromosomes of cancer cells. This technique has been applied in porcine cytogenetics in order to detect small chromosomal losses and detect aneuploidy in porcine embryos [143,144]. Array-CGH refines this technology using bacterial artificial chromosome (BAC) clone inserts, or short oligonucleotide sequences spaced over the entire chromosome or a region of interest to enhance resolution [145]. The primary application of array-CGH is the detection of disease-associated complex chromosome rearrangements, such as rearrangements associated with tumors. Array-CGH may also have applications in detecting large copy number variants (deletions and duplications) that may be associated with a specific phenotype or disease [146,147].

WGS methods such as Illumina next-generation sequencing provide a higher resolution look at the genome than DNA microarrays by fragmenting DNA into short DNA segments several bases to hundreds of bases long. Adapters are ligated to these DNA segments and then amplified via polymerase chain reaction (PCR), producing several copies of each DNA segment. The DNA segments are then exposed to fluorescently labelled nucleotides and DNA polymerase, binding to one base at a time, and taking an image that is interpreted by computer software. This process is repeated several times, allowing for each segment to be sequenced several times over and aligned, producing an accurate sequence of the genomic region of interest. WGS has been applied by cytogeneticists to delineate chromosome rearrangement breakpoints, and the breakpoint signatures coinciding with the repair mechanism, small copy number variants and indels (CNV less than 1 kb in length) not visible via SNP array, and novel single nucleotide variants within genes that may be associated with disease [148]. Although a relatively new technology, the cost of WGS in the pig has dropped dramatically in recent years, enabling methods such as Illumina short-read sequencing to be more widely implemented in research, facilitating the sequencing of several hundred pig genomes for variant discovery [149,150,151].

The application of WGS results in reads that provide the base pair sequence of regions of chromosomes, or whole chromosomes. WGS may detect balanced rearrangements using split alignments that map to two different locations in the reference genome, and discordant read pairs, paired ends that do not align within an expected distance or orientation [152]. These methods indicate regions of chromosomes where breakpoint junctions may occur and are the most successful and precise methods of identifying balanced rearrangements [153]. The most used sequencing methods for the detection of balanced reciprocal translocation in humans include long-read, short-read, and linked-read sequencing. Long-read sequencing is an expensive technique capable of producing long continuous sequences reads of DNA > 10 kb [154]. Long-read sequencing may be used to detect any type of structural variation; however, it is most successful at the detection of complex rearrangements, and breakpoints present in repetitive elements relative to other methods of WGS [153,155,156]. Short-read sequencing in contrast produces many overlapping short reads (150–300 bases) of the DNA [157]. Short-read sequencing is more established and less expensive than long-read sequencing, and is most useful for detecting simple rearrangements such as reciprocal translocations [158,159,160]. Linked-read sequencing is a method that uses small amounts of high-molecular-weight genomic DNA, spread across 100,000 droplets, each of which is tagged with a barcode [161]. The barcode-tagged droplets undergo short-read sequencing, with a computer algorithm using the barcode to link the sequenced reads to the original molecules and construct continuous segments of DNA. From here, structural variants may be determined from reads belonging to disparate regions but sharing the same barcode. Although less developed than long-read and short-read sequencing, linked-read sequencing has been successfully implemented in the detection of rearrangements spanning repetitive elements, overcoming one of the deficiencies of short-read sequencing [162].

In research, WGS has been used to detect a wide range of mutations, including copy number variations [163,164,165] as well as balanced chromosomal rearrangements such as translocations [148,166] and inversions [167]. Approximately 90% of breakpoints of balanced rearrangements can be identified using WGS [159]. Notably, low-level mosaicism and Robertsonian rearrangements, as well as supernumerary chromosomes and a subset of reciprocal translocations, especially those with breakpoint in repetitive sequences, cannot be routinely detected through short-read sequencing [159,168]. The cost of WGS, and the fact that not all rearrangements may be detected by WGS, indicates that cytogenomics are unlikely to overtake the classical and molecular cytogenetics techniques as a standard test for the detection of chromosome rearrangements. Even in the case of WGS being used to identify a rearrangement, it is recommended that follow-up studies consist of karyotyping and/or FISH in order to visualize the rearrangement and determine the structural rearrangement underlying the imbalance [169,170].

## 14. Implementation of Cytogenomics in Clinical Cytogenetics

The use of DNA microarrays such as SNP-array genotyping is widespread in the pig, and is primarily used for the purposes of genomic selection [136]. SNP-array genotyping and array-CGH are comparatively seldom applied in the field of clinical cytogenetics as both techniques are ineffective at identifying the balanced chromosome rearrangements characteristic of the pig and instead are only capable of detecting rare unbalanced rearrangements [171]. Instead, DNA microarrays are more often applied in order to identify copy number variants in pigs, deletions and duplications of genomic material that may be identified as they are characterized by genomic imbalance. Analysis of CNV rarely falls under the purview of cytogenetics laboratories as these structural variants are often too small to be detected through classical or molecular cytogenetics techniques. The importance of CNV has increased in recent years, with these structural variants being linked to much of the genomic variation observed in mammalian species and associated with diseases seen in human and animal genomes [172]. Currently, studies of CNV have sought to link these variants to traits relevant for breeding such as meat quality [173] and fertility [174], as well as diseases such as porcine reproductive and respiratory syndrome [175]. The expansion of WGS technologies will allow for more in-depth exploration of smaller structural variants in the genomes of pigs such as CNV and indels, along with an increased understanding of those variants with both economically desirable traits as well as disease. The linkage of CNV to fertility may indicate that one-day it may be routine to include screening for specific CNV alongside chromosome rearrangements in pigs prior to breeding [174].

SNP array genotyping has been used to identify the sire of unbalanced rearrangement carriers through identifying an imbalance in the inheritance of paternal alleles, resulting from a partial monosomy of chromosome 8, and a partial trisomy of chromosome 14 [3]. Although SNP genotypes may be used to identify unbalanced rearrangements and aneuploidies, such chromosomal events are rare in the pig, occurring in less than 10% of cases of clinically diagnosed chromosome abnormality [1]. The use of SNP array genotyping for the identification of balanced chromosome rearrangements may be applicable in a small subset of cases using karyomapping. This approach determines the linkage phase of SNPs, and has seen application as a pre-implantation genetic test for known rearrangement carriers, allowing those embryos with the same linkage phase as the rearrangement carrier to be identified [176]. This technique is unlikely to see widespread application in clinical cytogenetics programs as the carrier must be identified prior to karyomapping, and is only useful in cases where the goal is the identification of the offspring of carriers or of the parents of carriers.

WGS technologies provide the clearest avenue for implementation of cytogenomics techniques into clinical cytogenetics laboratories. Classical chromosome banding techniques as well as molecular technique such as FISH, although effective at identifying large rearrangements, may miss smaller terminal rearrangements and are incapable of identifying the precise breakpoints of the rearrangements [110]. The application of sequencing technologies will allow for further refinements to the identification and study of chromosome rearrangements, allowing for breakpoint junctions to be delineated. WGS has been successfully applied in humans to identify the precise breakpoint junctions of hundreds of balanced rearrangements [148,177]. Currently, WGS has been applied to just a handful of chromosome rearrangements in the pig, with the high cost of genome sequencing only recently reduced serving as a barrier to the more widespread implementation of WGS in the pig [126]. In one case, short-read sequencing was conducted on boars carrying unbalanced rearrangements [3]. The rearrangement was identified through reduced sequence coverage on chromosome 8, and increased sequence coverage on chromosome 14, corresponding to a partial monosomy and a partial trisomy, along with discordant paired-end sequence reads aligning on chromosomes 8 and 14, confirming the presence of a rearrangement [3].

The largest study of the applications of WGS into the study of porcine chromosome rearrangements performed short-read sequencing to seven carriers of karyotypically balanced rearrangements alongside 15 non-carriers [178]. Here, it was found that short-read sequencing was capable of accurately detecting the breakpoint junctions of six of seven carriers, with no false-positives detected. The main deficiency of short-read sequencing noted was that it was not capable of identifying breakpoint junctions occurring in repetitive sequences [178]. This study for the first time described the breakpoints of chromosome rearrangements in the pig genome, identifying several varieties of breakpoint signatures including microhomology, microinsertions, and blunt-end ligations also characteristic of human rearrangement breakpoint junctions [156,178]. Genes disrupted by the breakpoint interrupting the gene sequence were also found at the sites of breakpoint junctions, with the heterozygous nature of the disruption suggested to be protective of any phenotypic effect associated with the rearrangement. The sequencing of these breakpoints therefore indicates that so-called balanced rearrangements may not be as balanced as once thought, with the presence of small deletions, insertions, and gene disruptions noted as occurring in the pig [178].

The factors influencing the formation of chromosome rearrangements in pigs are still largely unknown, despite the sequencing of a handful of rearrangement breakpoint junctions. No clear pattern presents itself, with breakpoints appearing with different breakpoint signatures and in a variety of chromosome regions and landscapes [148,156,178]. A study of mosaic rearrangement carriers indicated that relatives of mosaic carriers themselves carried mosaic rearrangements at 2.5× the frequency as control animals, indicating a possible genetic component to the formation of chromosome rearrangements [74]. A preliminary genome-wide association study (GWAS) was performed in our laboratory on 15 carriers of reciprocal translocations, and 11 control boars with normal karyotypes, revealing the presence of five SNPs on three chromosomes associated with reciprocal translocation [179]. Functional analysis of these SNPs revealed that each was in close proximity (<2 Mb) to genes playing roles in the maintenance of DNA, detection of DNA damage, and the initiation of the DNA damage response [179]. These results indicate that genetic factors may play a large role in the susceptibility of pigs to produce de-novo chromosome rearrangements during meiotic events that are then passed on to offspring. The identification of SNPs closely linked to chromosome rearrangement could be incorporated alongside other cytogenomics analyses, as a control effort to identify boars at risk of producing carrier offspring.

## 15. Future Perspectives and Conclusions

The factors influencing the formation of chromosome rearrangements in mammalian genomes are still poorly understood. Various chromosomal characteristics such as relative chromosome density and the presence of common fragile sites have been associated with breakage hotspots in the pig genome [43]. The precise breakpoints, and the genomic landscape surrounding those breaks, is largely unknown, making it difficult to determine why a given chromosome region may experience more or less chromosome breaks and how this may subsequently lead to permanent rearrangement of chromosomes. The application of classical banding, and molecular cytogenetics techniques (although they have limited applications), will still be useful for primary identification of gross chromosome rearrangements. Therefore, it is necessary to incorporate WGS technologies into clinical cytogenetics programs in order to visualize rearrangements at the highest level, allowing for the delineation of breakpoints and the best understanding of any genomic consequences associated with the rearrangement.

Although currently too expensive for widespread application into clinical cytogenetics programs, the cost of WGS in humans has dramatically dropped in recent years from >$10,000 USD in 2010 to under $1000 USD as of 2020 [180]. Given this trend, it is not too unreasonable to predict that the price of DNA sequencing may further reduce in the coming years, facilitating the widespread introduction of WGS into the livestock industry. Access to sequencing data could allow for the routine detection of various chromosome rearrangements and aneuploidies ranging from balanced and unbalanced rearrangements, and CNV, to aneuploidies. This could be a cost-effective strategy for both breeders and cytogeneticists allowing for a wide range of accurate tests to be conducted on a single genomic data set. Although unlikely to fully replace banding techniques and FISH for chromosome analysis as both techniques allow for the visualization of the rearrangement, cost-reductions in WGS could enable DNA sequencing to be a first-tier genetic test for livestock [165,167].

The development and implementation of laboratory and genomic techniques for use in clinical cytogenetics has played an important role in swine breeding for the last forty years. From the first chromosome rearrangements and abnormalities identified, it has been clear that the pig is susceptible to a number of chromosome abnormalities resulting in impaired fertility to total infertility [181]. The development of the classical cytogenetics techniques and their subsequent application in pigs led to the first clinical cytogenetics programs, and to this day continue to form the basis of clinical cytogenetics operations. With the development of molecular cytogenetics techniques such as FISH, further refinements were made to the visualization of chromosome rearrangements, allowing rearrangements to be viewed at the highest resolution yet, along with refinements made to the identification of breakpoints, and the identification of sub-telomeric breaks. Lastly, as WGS technologies continue to be developed and applied in the pig, along with associated cost-reductions, there is an opportunity to revolutionize livestock breeding and clinical cytogenetics, allowing for DNA sequencing data to be used in concert with banding techniques or FISH to identify and study a large range of chromosomal rearrangements and abnormalities furthering the study and understanding of chromosome rearrangements. It is hopeful that with the implementation of WGS technologies, our understanding of chromosome rearrangements will increase and the factors influencing rearrangements in the pig genome be fully understood.

## References

[1] Raudsepp T., Chowdhary B.P. (2011). Cytogenetics and chromosome maps. the Genetics of the Pig.

[2] Gustavsson I. (1990). Chromosomes of the pig. Advances in Veterinary Science and Comparative Medicine.

[3] Grahofer A., Letko A., Häfliger I.M., Jagannathan V., Ducos A., Richard O., Peter V., Nathues H., Drögemüller C. (2019). Chromosomal imbalance in pigs showing a syndromic form of cleft palate. BMC Genom..

[4] Quach A.T., Revay T., Villagomez D.A.F., Macedo M.P., Sullivan A., Maignel L., Wyss S., Sullivan B., King W.A. (2016). Prevalence and consequences of chromosomal abnormalities in Canadian commercial swine herds. Genet. Sel. Evol..

[5] Danielak-Czech B., Kozubska-Sobocińska A., Rejduch B. (2016). Molecular Cytogenetics in the Diagnostics of Balanced Chromosome Mutations in the Pig (*Sus scrofa*)—A Review. Ann. Anim. Sci..

[6] King W.A., Gustavsson I., Popescu C.P., Linares T. (1981). Gametic products transmitted by rcp (13q−; 14q+) translocation heterozygous pigs, and resulting embryonic loss. Hereditas.

[7] Pinton A., Ducos A., Berland H., Seguela A., Brun-Baronnat C., Darré A., Darré R., Schmitz A., Yerle M. (2004). Chromosomal Abnormalities in Hypoprolific Boars. Hereditas.

[8] Ducos A., Berland H.-M., Bonnet N., Calgaro A., Billoux S., Mary N., Garnier-Bonnet A., Darré R., Pinton A. (2007). Chromosomal control of pig populations in France: 2002–2006 survey. Genet. Sel. Evol..

[9] King W.A., Donaldson B., Rezaei S., Schmidt C., Revay T., Villagomez D.A., Kuschke K., Moo-Young M. (2019). Chromosomal abnormalities in swine and their impact on production and profitability. Comprehensive Biotechnology.

[10] Ducos A., Berland H.M., Pinton A., Guillemot E., Seguela A., Blanc M.F., Darre A., Darre R. (1998). Nine new cases of reciprocal translocation in the domestic pig (*Sus scrofa domestica* L.). J. Hered..

[11] Ducos A., Revay T., Kovacs A., Hidas A., Pinton A., Bonnet-Garnier A., Molteni L., Slota E., Switonski M., Arruga M.V. (2008). Cytogenetic screening of livestock populations in Europe: An overview. Cytogenet. Genome Res..

[12] Bryden W. (1993). The chromosomes of the pig. Cytologia.

[13] Krallinger H.F. (1931). Cytologische Studien an Einigen Haussäugetieren [Cytological Studies on Some Domestic Animals].

[14] McConnell J., Fechheimer N.S., Gilmore L.O. (1963). Somatic Chromosomes of the Domestic Pig. J. Anim. Sci..

[15] Lejeune J. (1959). Etude des Chromosomes Somatiques de Neuf Enfants Mongoliens [Study of the Somatic Chromosomes of Nine Mongoloid Children]. CR Acad. Sci..

[16] Patau K., Smith D., Therman E., Inhorn S., Wagner H. (1960). Multiple congenital anomaly caused by an extra autosome. Lancet.

[17] Edwards J., Harnden D., Cameron A., Crosse V., Wolf O. (1960). A new trisomic syndrome. Lancet.

[18] McIlree M., Price W., Brown W., Tulloch W., Newsam J., MacLean N. (1966). sChromosome studies on testicular cells from 50 subfertile men. Lancet.

[19] Philip J., Skakkebæk N.E., Hammen R., Johnsen S.G., Rebbe H. (1970). Cytogenetic investigations in male infertility. Acta Obstet. Gynecol. Scand..

[20] Chandley A.C., Edmond P., Christie S., Gowans L., Fletcher J., Frackiewicz A., Newton M. (1975). Cytogenetics and infertility in man. I. Karyotype and seminal analysis: Results of a five-year survey of men attending a subfertility clinic. Ann. Hum. Genet..

[21] Moorhead P.S., Nowell P.C., Mellman W.J., Battips D.T., Hungerford D.A. (1960). Chromosome preparations of leukocytes cultured from human peripheral blood. Exp. Cell Res..

[22] Arakaki D., Sparkes R. (1963). Microtechnique for Culturing Leukocytes from Whole Blood. Cytogenet. Genome Res..

[23] Caspersson T., Zech L., Johansson C., Modest E.J. (1970). Identification of human chromosomes by DNA-binding fluorescent agents. Chromosoma.

[24] Seabright M. (1971). A rapid banding technique for human chromosomes. Lancet.

[25] Dutrillaux B., Lejeune J. (1971). Sur une Nouvelle Technique D’analyse du Caryotype Humain [On a New Technique for Analyzing the Human Karyotype]. CR Acad. Sci..

[26] Dutrillaux B. (1973). Coloration des Chromosomes Humains par L’acridine Orange Après Traitement par le 5 Bromodéoxyuridine [Staining of Human Chromosomes with Acridine Orange after Treatment with 5 Bromodeoxyuridine]. CR Acad. Sci..

[27] Wang H.C., Fedoroff S. (1972). Banding in Human Chromosomes treated with Trypsin. Nat. New Biol..

[28] Bickmore A.W. (2001). Karyotype analysis and chromosome banding. eLS.

[29] Hageltorn M., Gustavsson I. (2009). Giemsa staining patterns for identification of the pig mitotic chromosomes. Hereditas.

[30] Gustavsson I. (1980). Banding techniques in chromosome analysis of domestic animals. Adv. Vet. Sci. Comp. Med..

[31] Sumner A. (1972). A simple technique for demonstrating centromeric heterochromatin. Exp. Cell Res..

[32] Bloom S.E., Goodpasture C. (1976). An improved technique for selective silver staining of nucleolar organizer regions in human chromosomes. Qual. Life Res..

[33] Dutrillaux B. (1973). Nouveau Système de Marquage Chromosomique: Les Bandes T [New Chromosome Labeling System: T Bands]. Chromosoma.

[34] Gustavsson I. (1988). Standard karyotype of the domestic pig: Committee for the Standardized Karyotype of the Domestic Pig. Hereditas.

[35] Vorsanova S.G., Yurov Y.B., Iourov I.Y. (2010). Human interphase chromosomes: A review of available molecular cytogenetic technologies. Mol. Cytogenet..

[36] Berardino D.D., Lannuzzi L., Lioi M. (1985). The high-resolution RBA-banding pattern of bovine chromosomes. Cytogenet. Genome Res..

[37] Rønne M. (1990). Chromosome preparation and high resolution banding. In Vivo.

[38] Yerle M., Galman O., Echard G. (1991). The high-resolution GTG-banding pattern of pig chromosomes. Cytogenet. Genome Res..

[39] Gustavsson I., Hageltorn M., Johansson C., Zech L. (1972). Identification of the pig chromosomes by the quinacrine mustard fluorescence technique. Exp. Cell Res..

[40] Lin C.C., Biederman B.M., Jamro H.K., Hawthorne A.B., Church R.B. (1980). Porcine (*Sus scrofa domestica*) chromosome identification and suggested nomenclature. Can. J. Genet. Cytol..

[41] Rønne M., Stefanova V., Di Berardino D., Poulsen B.S. (2008). The R-banded karyotype of the domestic pig (*Sus scrofa dornestica* L.). Hereditas.

[42] Ford C.E., Pollock D.L., Gustavsson I. (1980). Proceedings of the First International Conference for the Standardisation of Banded Karyotypes of Domestic Animals University of Reading Reading, England, 2–6 August 1976. Hereditas.

[43] Donaldson B., Villagomez D.A., Revay T., Rezaei S., King W.A. (2019). Non-Random distribution of reciprocal translocation breakpoints in the pig genome. Genes.

[44] Sánchez-Sánchez R., Gómez-Fidalgo E., Pérez-Garnelo S., Martín-Lluch M., De La Cruz-Vigo P. (2019). Prevalence of chromosomal aberrations in breeding pigs in Spain. Reprod. Domest. Anim..

[45] Basrur P., Stranzinger G. (2008). Veterinary cytogenetics: Past and perspective. Cytogenet. Genome Res..

[46] Dagorn R. (1978). Note Aux Établissements Départementaux de l’Elevage.

[47] Popescu C.P., Boscher J., Tixier M. (1983). Une nouvelle translocation réciproque t, rcp (7q−; 15q+) chez un verrat «hypoprolifique» [A new reciprocal translocation, rcp (7q−; 15q+) translocation in a “hypoprolific” boar]. Génétique Sél. Évol..

[48] Ducos A., Pinton A., Berland H.-M., Seguela A., Blanc M.-F., Darre A., Darre R. (2004). Five New Cases of Reciprocal Translocation in the Domestic Pig. Hereditas.

[49] Ducos A., Pinton A., Yerle M., Séguéla A., Berland H.-M., Brun-Baronnat C., Bonnet N., Darré R. (2002). Cytogenetic and molecular characterization of eight new reciprocal translocations in the pig species. Estimation of their incidence in French populations. Genet. Sel. Evol..

[50] Ducos A., Calgaro A., Mouney-Bonnet N., Loustau A.M., Revel C., Barasc H., Mary N., Pinton A. (2017). Chromosomal control of pig populations in France: A 20-year perspective. Journées Rech. Porc. Fr..

[51] Pinton A., Calgaro A., Bonnet N., Mary N., Dudez A.M., Barasc H., Plard C., Yerle M., Ducos A. (2012). Chromosomal control of pig populations in France: 2007–2010 survey. Journées Rech. Porc. Franc..

[52] Villagómez D.A., Revay T., Donaldson B., Rezaei S., Pinton A., Palomino M., Junaidi A., Honaramooz A., King W.A. (2017). Azoospermia and Testicular Hypoplasia in a Boar Carrier of a Novel Y-Autosome Translocation. Sex. Dev..

[53] Tikhonov V.N., Troshina A.I. (1975). Chromosome translocations in the karyotypes of wild boars *Sus scrofa* L. of the European and the Asian areas of USSR. Theor. Appl. Genet..

[54] Rejduch B., Slota E., Rozycki M., Koscielny M. (2003). Chromosome number polymorphism in a litter of European wild boar (*Sus scrofa scrofa* L.). Anim. Sci. Pap. Rep..

[55] Miyake Y.-I., Kawata K., Ishikawa T., Umezu M. (1977). Translocation heterozygosity in a malformed piglet and its normal littermates. Teratology.

[56] Alonso R.A., Cantu J.M. (1982). A Robertsonian translocation in the domestic pig (*Sus scrofa*) 37,XX,-13,-17,t rob(13;17). Ann. Génétique.

[57] Schwerin M., Golisch D., Ritter E. (1986). A Robertsonian translocation in swine. Genet. Sel. Evol..

[58] Danielak-Czech B., Słota E. (2007). A new case of reciprocal translocation t (10;13) (q16;q21) diagnosed in an AI boar. J. Appl. Genet..

[59] Pinton A., Calgaro A., Bonnet N., Ferchaud S., Billoux S., Dudez A., Mary N., Massip K., Bonnet-Garnier A., Yerle M. (2009). Influence of sex on the meiotic segregation of a t (13;17) Robertsonian translocation: A case study in the pig. Hum. Reprod..

[60] Switonski M., Stranzinger G. (1998). Studies of synaptonemal complexes in farm mammals—A review. J. Hered..

[61] Villagómez D., Pinton A. (2008). Chromosomal abnormalities, meiotic behavior and fertility in domestic animals. Cytogenet. Genome Res..

[62] McFeely R.A. (1996). A direct method for the display of chromosomes from early pig embryos. Reproduction.

[63] Lojda L. (1975). The cytogenetic pattern in pigs with hereditary intersexuality similar to the syndrome of testicular feminization in man. Acta Vet. Brno.

[64] Breeuwsma A.J. (1968). A case of XXY sex chromosome constitution in an intersex pig. Reproduction.

[65] Hancock J.L., Daker M.G. (1981). Testicular hypoplasia in a boar with abnormal sex chromosome constitution (39 XXY). Reproduction.

[66] Mäkinen A., Andersson M., Nikunen S. (1998). Detection of the X chromosomes in a Klinefelter boar using a whole human X chromosome painting probe. Anim. Reprod. Sci..

[67] Ducos A., Berland H.M., Pinton A., Calgaro A., Brun-Baronnat C., Bonnet N., Garnier-Bonnet A., Darré R. (2004). Chromosome control of domestic animal populations in France. 16th European Colloquium on Animal Cytogenetics and Gene Mapping. Cytogenet. Genome Res..

[68] Villagómez D.A.F., Gustavsson I., Jönsson L., Plöen L. (2004). Reciprocal Chromosome Translocation, rcp(7;17)(q26;q11), in a Boar Giving Reduced Litter Size and Increased Rate of Piglets Dying in the Early Life. Hereditas.

[69] Rezaei S., Donaldson B., Villagomez D.A.F., Revay T., Mary N., Grossi D.A., King W.A. (2020). Routine Karyotyping Reveals Frequent Mosaic Reciprocal Chromosome Translocations in Swine: Prevalence, Pedigree, and Litter Size. Sci. Rep..

[70] Musilova P., Drbalova J., Kubickova S., Cernohorska H., Stepanova H., Rubes J. (2014). Illegitimate recombination between T cell receptor genes in humans and pigs (*Sus scrofa domestica*). Chromosom. Res..

[71] Quilter C.R., Wood D., Southwood O.I., Griffin D.K. (2003). X/XY/XYY mosaicism as a cause of subfertility in boars: A single case study. Anim. Genet..

[72] Bruère A., Fielden E., Hutchings H. (1968). XX/XY mosaicism in lymphocyte cultures from a pig with freemartin characteristics. N. Z. Vet. J..

[73] Somlev B., Hansen-Melander E., Melander Y., Holm L. (2009). XX/XY chimerism in leucocytes of two intersexual pigs. Hereditas.

[74] Toyama Y. (1974). Sex chromosome mosaicisms in five swine intersexes. Jpn. J. Zootech. Sci..

[75] Christensen K., Nielsen P.B. (1980). A case of blood chimerism (XX, XY) in pigs. Anim. Blood Groups Biochem. Genet..

[76] Clarkson B.G., Fisher K.R.S., Partlow G.D. (1995). Agonadal presumptive XX/XY leukochimeric pig. Anat. Rec. Adv. Integr. Anat. Evol. Biol..

[77] Tsai A.G., Lieber M.R. (2010). Mechanisms of chromosomal rearrangement in the human genome. BMC Genom..

[78] Hiraiwa H., Uenishi H., Kiuchi S., Watanabe M., Takagaki Y., Yasue H. (2001). Assignment of T cell receptor (TCR) alpha-chain gene (A), beta-chain gene (B), gamma-chain gene (G), and delta-chain gene (D) loci on swine chromosomes by in situ hybridization and radiation hybrid mapping. Cytogenet. Cell Genet..

[79] Riggs P., Kuczek T., Chrisman C., Bidwell C. (1993). Analysis of aphidicolin-induced chromosome fragility in the domestic pig (*Sus scrofa*). Cytogenet. Genome Res..

[80] Yang M., Long S. (1993). Folate sensitive common fragile sites in chromosomes of the domestic pig (*Sus scrofa*). Res. Vet. Sci..

[81] Rønne M. (2004). Localization of Fragile Sites in the Karyotype of *Sus scrofa domestica*: Present Status. Hereditas.

[82] Riggs P., Rønne M. (2009). Fragile Sites in Domestic Animal Chromosomes: Molecular Insights and Challenges. Cytogenet. Genome Res..

[83] Rubeš J., Pinton A., Bonnet-Garnier A., Fillon V., Musilova P., Michalova K., Kubíčková S., Ducos A., Yerle M. (2009). Fluorescence in situ Hybridization Applied to Domestic Animal Cytogenetics. Cytogenet. Genome Res..

[84] Pinkel D., Straume T., Gray J.W. (1986). Cytogenetic analysis using quantitative, high-sensitivity, fluorescence hybridization. Proc. Natl. Acad. Sci. USA.

[85] Mao S.Y., Mullins J.M. (2010). Conjugation of fluorochromes to antibodies. Immunocytochemical Methods and Protocols.

[86] Fahrenkrug S.C., Rohrer G.A., Freking B.A., Smith T.P., Osoegawa K., Shu C.L., Catanese J.J., De Jong P.J. (2001). A porcine BAC library with tenfold genome coverage: A resource for physical and genetic map integration. Mamm. Genome.

[87] Schmitz A., Chaput B., Fouchet P., Guilly M.N., Frelat G., Vaiman M. (1992). Swine chromosomal DNA quantification by bivariate flow karyotyping and karyotype interpretation. Cytom. J. Int. Soc. Anal. Cytol..

[88] Telenius H., Ponder B.A.J., Tunnacliffe A., Pelmear A.H., Carter N.P., Ferguson-Smith M.A., Behmel A., Nordenskjöld M., Pfragner R. (1992). Cytogenetic analysis by chromosome painting using dop-pcr amplified flow-sorted chromosomes. Genes Chromosom. Cancer.

[89] Langford C.F., Telenius H., Miller N.G.A., Thomsen P.D., Tucker E.M. (2009). Preparation of chromosome-specific paints and complete assignment of chromosomes in the pig flow karyotype. Anim. Genet..

[90] Scalenghe F., Turco E., Edström J.E., Pirrotta V., Melli M. (1981). Microdissection and cloning of DNA from a specific region of Drosophila melanogaster polytene chromosomes. Chromosoma.

[91] Chaudhary R., Kijas J., Raudsepp T., Guan X.Y., Zhang H., Chowdhary B.P. (1998). Microdissection of pig chromosomes: Dissection of whole chromosomes, arms and bands for construction of paints and libraries. Hereditas.

[92] Pinton A., Ducos A., Yerle M. (2003). Chromosomal rearrangements in cattle and pigs revealed by chromosome microdissection and chromosome painting. Genet. Sel. Evol..

[93] Schermelleh L., Thalhammer S., Heckl W., Pösl H., Cremer T., Schütze K., Cremer M. (1999). Laser Microdissection and Laser Pressure Catapulting for the Generation of Chromosome-Specific Paint Probes. Biotechniques.

[94] Kubickova S., Cernohorska H., Musilova P., Rubes J. (2002). The use of laser microdissection for the preparation of chromosome-specific painting probes in farm animals. Chromosom. Res..

[95] Ried T., Schröck E., Ning Y., Wienberg J. (1998). Chromosome painting: A useful art. Hum. Mol. Genet..

[96] Koch J., Hindkjaer J., Mogensen J., Kølvraa S., Bolund L. (1991). An improved method for chromosome-specific labeling of α satellite DNA in situ by using denatured double-stranded DNA probes as primers in a primed in situ labeling (PRINS) procedure. Genet. Anal. Biomol. Eng..

[97] Seña C.D.L., Chowdhary B.P., Gustavsson I. (1995). Localization of the telomeric (TTAGGG) n sequences in chromosomes of some domestic animals by fluorescence in situ hybridization. Hereditas.

[98] Gu F., Hindkjaer J., Gustavsson I., Bolund L. (1996). A signal of telomeric sequences on porcine chromosome 6q21–q22 detected by primed in situ labelling. Chromosom. Res..

[99] Pellestor F., Girardet A., Lefort G., Andréo B., Charlieu J.P. (1995). Use of the primed in situ labelling (PRINS) technique for a rapid detection of chromosomes 13, 16, 18, 21, X and Y. Hum. Genet..

[100] Miller J.R., Hindkjaer J., Thomsen P.D. (1993). A chromosomal basis for the differential organization of a porcine centromere-specific repeat. Cytogenet. Genome Res..

[101] Rogel-Gaillard C., Bourgeaux N., Save J.C., Renard C., Coullin P., Pinton P., Yerle M., Vaiman M., Chardon P. (1997). Construction of a swine YAC library allowing an efficient recovery of unique and centromeric repeated sequences. Mamm. Genome.

[102] Danielak-Czech B., Rejduch B., Kozubska-Sobocińska A. (2013). Identification of telomeric sequences in pigs with rearranged karyotype using PRINS technique. Ann. Anim. Sci..

[103] Goureau A., Yerle M., Schmitz A., Riquet J., Milan D., Pinton P., Frelat G., Gellin J. (1996). Human and porcine correspondence of chromosome segments using bidirectional chromosome painting. Genomics.

[104] Chowdhary B.P., Raudsepp T., Frönicke L., Scherthan H. (1998). Emerging patterns of comparative genome organization in some mammalian species as revealed by Zoo-FISH. Genome Res..

[105] Sarrate Z., Anton E. (2009). Fluorescence in situ hybridization (FISH) protocol in human sperm. J. Vis. Exp. JoVE.

[106] Konfortova G., Miller N., Tucker E. (1995). A new reciprocal translocation (7q+;15q−) in the domestic pig. Cytogenet. Genome Res..

[107] Pinton A., Faraut T., Yerle M., Gruand J., Pellestor F., Ducos A. (2005). Comparison of male and female meiotic segregation patterns in translocation heterozygotes: A case study in an animal model (*Sus scrofa domestica* L.). Hum. Reprod..

[108] Pinton A., Ducos A., Séguéla A., Berland H.M., Darré R., Darré A., Pinton P., Schmitz A., Cribiu E.P., Yerle M. (1998). Characterization of reciprocal translocations in pigs using dual-colour chromosome painting and primed in situ DNA labelling. Chromosome Res..

[109] Pinton A., Pailhoux E., Piumi F., Rogel-Gaillard C., Darré R., Yerle M., Ducos A., Cotinot C. (2002). A case of intersexuality in pigs associated with a de novo paracentric inversion 9 (p1. 2; p2. 2). Anim. Genet..

[110] O’Connor R.E., Fonseka G., Frodsham R., Archibald A.L., Lawrie M., Walling G.A., Griffin D.K. (2017). Isolation of subtelomeric sequences of porcine chromosomes for translocation screening reveals errors in the pig genome assembly. Anim. Genet..

[111] Barasc H., Mary N., Letron R., Calgaro A., Dudez A., Bonnet N., Lahbib-Mansais Y., Yerle M., Ducos A., Pinton A. (2012). Y-Autosome Translocation Interferes with Meiotic Sex Inactivation and Expression of Autosomal Genes: A Case Study in the Pig. Sex. Dev..

[112] Mary N., Barasc H., Ferchaud S., Billon Y., Meslier F., Robelin D., Calgaro A., Loustau-Dudez A.M., Bonnet N., Yerle M. (2014). Meiotic recombination analyses of individual chromosomes in male domestic pigs (*Sus scrofa domestica*). PLoS ONE.

[113] Pinton A., Letron I.R., Berland H., Bonnet N., Calgaro A., Garnier-Bonnet A., Yerle M., Ducos A. (2008). Meiotic studies in an azoospermic boar carrying a Y;14 translocation. Cytogenet. Genome Res..

[114] Bonnet-Garnier A., Guardia S., Pinton A., Ducos A., Yerle M. (2009). Analysis using sperm-FISH of a putative interchromosomal effect in boars carrying reciprocal translocations. Cytogenet. Genome Res..

[115] Massip K., Bonnet N., Calgaro A., Billoux S., Baquié V., Mary N., Bonnet-Garnier A., Ducos A., Yerle M., Pinton A. (2009). Male Meiotic Segregation Analyses of Peri- and Paracentric Inversions in the Pig Species. Cytogenet. Genome Res..

[116] Massip K., Yerle M., Billon Y., Ferchaud S., Bonnet N., Calgaro A., Mary N., Dudez A.-M., Sentenac C., Plard C. (2010). Studies of male and female meiosis in inv (4) (p1.4; q2.3) pig carriers. Chromosom. Res..

[117] Danielak-Czech B., Kozubska-Sobocińska A., Rejduch B. (2010). Diagnosis of tandem fusion translocation in the boar using FISH technique with human painting probes. Ann. Anim. Sci..

[118] Rejduch B., Slota E., Sysa P., Koscielny M., Wrzeska M., Babicz M. (2003). Synaptonemal complexes analysis of the European wild boars û carriers of the 15; 17 Robertsonian translocation. Rocz. Nauk. Zootech..

[119] Kawarasaki T., Matsumoto K., ChiKyu M., Itagaki Y., Horiuchi A., Murofushi J. (2000). Sexing of porcine embryo by in situ hybridization using chromosome Y- and 1-specific DNA probes. Theriogenology.

[120] Parrilla I., Vázquez J.M., Oliver-Bonet M., Navarro J., Yelamos J., Roca J., Martínez E.A. (2003). Fluorescence in situ hybridization in diluted and flow cytometrically sorted boar spermatozoa using specific DNA direct probes labelled by nick translation. Reprod. Camb..

[121] Rubeš J., Vozdova M., Kubíčková S. (1999). Aneuploidy in pig sperm: Multicolor fluorescence in situ hybridization using probes for chromosomes 1, 10, and Y. Cytogenet. Genome Res..

[122] Pinton A., Ducos A., Yerle M. (2004). Estimation of the proportion of genetically unbalanced spermatozoa in the semen of boars carrying chromosomal rearrangements using FISH on sperm nuclei. Genet. Sel. Evol..

[123] Massip K., Berland H., Bonnet N., Calgaro A., Billoux S., Baquié V., Mary N., Bonnet-Garnier A., Ducos A., Yerle M. (2008). Study of inter- and intra-individual variation of meiotic segregation patterns in t (3;15) (q27;q13) boars. Theriogenology.

[124] Russo C.D., Di Giacomo G., Cignini P., Padula F., Mangiafico L., Mesoraca A., D’Emidio L., McCluskey M.R., Paganelli A., Giorlandino C. (2014). Comparative study of aCGH and Next Generation Sequencing (NGS) for chromosomal microdeletion and microduplication screening. J. Prenat. Med..

[125] Crosetto N., Mitra A., Silva M.J., Bienko M., Dojer N., Wang Q., Karaca E., Chiarle R., Skrzypczak M., Ginalski K. (2013). Nucleotide-resolution DNA double-strand break mapping by next-generation sequencing. Nat. Methods.

[126] Warr A., Affara N., Aken B., Beiki H., Bickhart D.M., Billis K., Chow W., Eory L., Finlayson H.A., Flicek P. (2020). An improved pig reference genome sequence to enable pig genetics and genomics research. GigaScience.

[127] Servin B., Faraut T., Iannuccelli N., Zelenika D., Milan D. (2012). High-Resolution autosomal radiation hybrid maps of the pig genome and their contribution to the genome sequence assembly. BMC Genom..

[128] Tortereau F., Servin B., Frantz L., Megens H.J., Milan D., Rohrer G., Wiedmann R., Beever J., Archibald A.L., Schook L.B. (2012). A high density recombination map of the pig reveals a correlation between sex-specific recombination and GC content. BMC Genom..

[129] Yerle M., Lahbib-Mansais Y., Mellink C., Goureau A., Pinton P., Echard G., Gellin J., Zijlstra C., De Haan N., Bosma A.A. (1995). The PiGMaP consortium cytogenetic map of the domestic pig (*Sus scrofa domestica*). Mamm. Genome.

[130] Humphray S.J., Scott C.E., Clark R., Marron B., Bender C., Camm N., Davis J., Jenks A., Noon A., Patel M. (2007). A high utility integrated map of the pig genome. Genome Biol..

[131] Groenen M.A., Archibald A.L., Uenishi H., Tuggle C.K., Takeuchi Y., Rothschild M.F., Schook L.B. (2012). Analyses of pig genomes provide insight into porcine demography and evolution. Nature.

[132] Ramos A.M., Crooijmans R.P.M.A., Affara N.A., Amaral A.J., Archibald A.L., Beever J.E., Bendixen C., Churcher C., Clark R., Dehais P. (2009). Design of a High Density SNP Genotyping Assay in the Pig Using SNPs Identified and Characterized by Next Generation Sequencing Technology. PLoS ONE.

[133] Hu Z.-L., Park C.A., Reecy J.M. (2016). Developmental progress and current status of the Animal QTLdb. Nucleic Acids Res..

[134] Bumgarner R. (2013). Overview of DNA microarrays: Types, applications, and their future. Curr. Protoc. Mol. Biol..

[135] Meuwissen T., Hayes B., Goddard M. (2013). Accelerating Improvement of Livestock with Genomic Selection. Annu. Rev. Anim. Biosci..

[136] Ibáñez-Escriche N., Forni S., Noguera J.L., Varona L. (2014). Genomic information in pig breeding: Science meets industry needs. Livest. Sci..

[137] Meuwissen T.H., Hayes B.J., Goddard M.E. (2001). Prediction of total genetic value using genome-wide dense marker maps. Genetics.

[138] Robinson J., Buhr M. (2005). Impact of genetic selection on management of boar replacement. Theriogenology.

[139] Christensen O., Madsen P., Nielsen B., Ostersen T., Su G. (2012). Single-step methods for genomic evaluation in pigs. Animal.

[140] Lee Y.-S., Shin D. (2018). Genome-Wide Association Studies Associated with Backfat Thickness in Landrace and Yorkshire Pigs. Genom. Inform..

[141] Verardo L.L., Sevón-Aimonen M.-L., Serenius T., Hietakangas V., Uimari P. (2017). Whole-genome association analysis of pork meat pH revealed three significant regions and several potential genes in Finnish Yorkshire pigs. BMC Genet..

[142] Wang Y., Ding X., Tan Z., Ning C., Xing K., Yang T., Pan Y., Sun D., Wang C. (2017). Genome-Wide Association Study of Piglet Uniformity and Farrowing Interval. Front. Genet..

[143] Apiou F., Vincent-Naulleau S., Spatz A., Vielh P., Geffrotin C., Frelat G., Dutrillaux B., Le Chalony C. (2004). Comparative genomic hybridization analysis of hereditary swine cutaneous melanoma revealed loss of the swine 13q36-49 chromosomal region in the nodular melanoma subtype. Int. J. Cancer.

[144] Horňák M., Hulinska P., Musilova P., Kubíčková S., Rubeš J. (2009). Investigation of Chromosome Aneuploidies in Early Porcine Embryos Using Comparative Genomic Hybridization. Cytogenet. Genome Res..

[145] Shinawi M., Cheung S.W. (2008). The array CGH and its clinical applications. Drug Discov. Today.

[146] Redon R., Ishikawa S., Fitch K.R., Feuk L., Perry G.H., Andrews T.D., Fiegler H., Shapero M.H., Carson A.R., Chen W. (2006). Global variation in copy number in the human genome. Nature.

[147] Fadista J., Nygaard M., Holm L.-E., Thomsen B., Bendixen C. (2008). A Snapshot of CNVs in the Pig Genome. PLoS ONE.

[148] Nilsson D., Pettersson M., Gustavsson P., Förster A., Hofmeister W., Wincent J., Zachariadis V., Anderlid B.-M., Nordgren A., Mäkitie O. (2017). Whole-Genome Sequencing of Cytogenetically Balanced Chromosome Translocations Identifies Potentially Pathological Gene Disruptions and Highlights the Importance of Microhomology in the Mechanism of Formation. Hum. Mutat..

[149] Li M., Chen L., Tian S., Lin Y., Tang Q., Zhou X., Li D., Yeung C.K.L., Che T., Li X. (2017). Comprehensive variation discovery and recovery of missing sequence in the pig genome using multiple de novo assemblies. Genome Res..

[150] Frantz L.A.F., Schraiber J.G., Madsen O.D., Megens H.-J., Cagan A., Bosse M., Paudel Y., Crooijmans R.P.M.A., Larson G., Groenen M.A.M. (2015). Evidence of long-term gene flow and selection during domestication from analyses of Eurasian wild and domestic pig genomes. Nat. Genet..

[151] Groenen M.A.M. (2016). A decade of pig genome sequencing: A window on pig domestication and evolution. Genet. Sel. Evol..

[152] Liu B., Conroy J.M., Morrison C.D., Odunsi A.O., Qin M., Wei L., Trump D.L., Johnson C.S., Liu S., Wang J. (2015). Structural variation discovery in the cancer genome using next generation sequencing: Computational solutions and perspectives. Oncotarget.

[153] Ho S.S., Urban A.E., Mills R.E. (2019). Structural variation in the sequencing era. Nat. Rev. Genet..

[154] Pollard M.O., Gurdasani D., Mentzer A.J., Porter T., Sandhu M.S. (2018). Long reads: Their purpose and place. Hum. Mol. Genet..

[155] Chow J.F., Cheng H.H., Lau E.Y., Yeung W.S., Ng E.H. (2020). Distinguishing between carrier and noncarrier embryos with the use of long-read sequencing in preimplantation genetic testing for reciprocal translocations. Genomics.

[156] Hu L., Liang F., Cheng D., Zhang Z., Yu G., Zha J., Wang Y., Xia Q., Yuan D., Tan Y. (2020). Location of Balanced Chromosome-Translocation Breakpoints by Long-Read Sequencing on the Oxford Nanopore Platform. Front. Genet..

[157] Goodwin S., McPherson J.D., McCombie W.R. (2016). Coming of age: Ten years of next-generation sequencing technologies. Nat. Rev. Genet..

[158] Dong Z., Jiang L., Yang C., Hu H., Wang X., Chen H., Choy K.W., Hu H., Dong Y., Hu B. (2014). A Robust Approach for Blind Detection of Balanced Chromosomal Rearrangements with Whole-Genome Low-Coverage Sequencing. Hum. Mutat..

[159] Redin C., Brand H., Collins R.L., Kammin T., Mitchell E., Hodge J.C., Hanscom C., Pillalamarri V., Seabra C.M., Abbott M.-A. (2017). The genomic landscape of balanced cytogenetic abnormalities associated with human congenital anomalies. Nat. Genet..

[160] Talkowski M.E., Ernst C., Heilbut A., Chiang C., Hanscom C., Lindgren A., Kirby A., Liu S., Muddukrishna B., Ohsumi T.K. (2011). Next-Generation Sequencing Strategies Enable Routine Detection of Balanced Chromosome Rearrangements for Clinical Diagnostics and Genetic Research. Am. J. Hum. Genet..

[161] Zheng G.X.Y., Lau B.T., Schnall-Levin M., Jarosz M., Bell J.M., Hindson C.M., Kyriazopoulou-Panagiotopoulou S., Masquelier D.A., Merrill L., Terry J.M. (2016). Haplotyping germline and cancer genomes with high-throughput linked-read sequencing. Nat. Biotechnol..

[162] Uguen K., Jubin C., Duffourd Y., Bardel C., Malan V., Dupont J.M., Khattabi L.E., Chatron N., Vitobello A., Sanlaville D. (2020). Genome sequencing in cytogenetics: Comparison of short-read and linked-read approaches for germline structural variant detection and characterization. Mol. Genet. Genom. Med..

[163] Gross A.M., Ajay S.S., Rajan V., Brown C., Bluske K., Burns N.J., Chawla A., Coffey A.J., Malhotra A., Scocchia A. (2019). Copy-Number variants in clinical genome sequencing: Deployment and interpretation for rare and undiagnosed disease. Genet. Med..

[164] Trost B., Walker S., Wang Z., Thiruvahindrapuram B., MacDonald J.R., Sung W.W., Pereira S.L., Whitney J., Chan A.J.S., Scherer S.W. (2018). A comprehensive workflow for read depth-based identification of copy-number variation from whole-genome sequence data. Am. J. Hum. Genet..

[165] Ellingford J.M., Campbell C., Barton S., Bhaskar S., Gupta S., Taylor R.L., Sergouniotis P.I., Horn B., Lamb J.A., Michaelides M. (2017). Validation of copy number variation analysis for next-generation sequencing diagnostics. Eur. J. Hum. Genet..

[166] Bramswig N.C., Lüdecke H.J., Pettersson M., Albrecht B., Bernier R.A., Cremer K., Eichler E.E., Falkenstein D., Gerdts J., Wieczorek D. (2017). Identification of new TRIP12 variants and detailed clinical evaluation of individuals with non-syndromic intellectual disability with or without autism. Hum. Genet..

[167] Grigelioniene G., Nevalainen P.I., Reyes M., Thiele S., Tafaj O., Molinaro A., Takatani R., Ala-Houhala M., Nilsson D., Jüppner H. (2017). A large inversion involving GNAS exon A/B and all exons encoding Gsα is associated with autosomal dominant pseudohypoparathyroidism type Ib (PHP1B). J. Bone Miner. Res..

[168] Hochstenbach R., Liehr T., Hastings R.J. (2021). Chromosomes in the genomic age. Preserving cytogenomic competence of diagnostic genome laboratories. Eur. J. Hum. Genet..

[169] Nowakowska B.A., De Leeuw N., Al Ruivenkamp C., Sikkema-Raddatz B., Crolla J.A., Thoelen R., Koopmans M., Hollander N.D., Van Haeringen A., Van Der Kevie-Kersemaekers A.-M. (2011). Parental insertional balanced translocations are an important cause of apparently de novo CNVs in patients with developmental anomalies. Eur. J. Hum. Genet..

[170] Silva M., De Leeuw N., Mann K., Schuring-Blom H., Morgan S., Giardino D., Rack K., Hastings R. (2018). European guidelines for constitutional cytogenomic analysis. Eur. J. Hum. Genet..

[171] Treff N.R., Tao X., Schillings W.J., Bergh P.A., Scott R.T., Levy B. (2011). Use of single nucleotide polymorphism microarrays to distinguish between balanced and normal chromosomes in embryos from a translocation carrier. Fertil. Steril..

[172] Zhang F., Gu W., Hurles M.E., Lupski J.R. (2009). Copy Number Variation in Human Health, Disease, and Evolution. Annu. Rev. Genom. Hum. Genet..

[173] Wang L., Xu L., Liu X., Zhang T., Li N., Hay E.H., Zhang Y., Yan H., Zhao K., Liu G.E. (2015). Copy number variation-based genome wide association study reveals additional variants contributing to meat quality in Swine. Sci. Rep..

[174] Revay T., Quach A.T., Maignel L., Sullivan B., King W.A. (2015). Copy number variations in high and low fertility breeding boars. BMC Genom..

[175] Hay E.H.A., Choi I., Xu L., Zhou Y., Rowland R.R.R., Lunney J.K., Liu G.E. (2017). CNV Analysis of Host Responses to Porcine Reproductive and Respiratory Syndrome Virus Infection. J. Genom..

[176] Handyside A.H., Harton G.L., Mariani B., Thornhill A.R., Affara N., Shaw M.-A., Griffin D.K. (2009). Karyomapping: A universal method for genome wide analysis of genetic disease based on mapping crossovers between parental haplotypes. J. Med. Genet..

[177] Luukkonen T.M., Mehrjouy M.M., Pöyhönen M., Anttonen A.K., Lahermo P., Ellonen P., Paulin L., Tommerup N., Palotie A., Varilo T. (2018). Breakpoint mapping and haplotype analysis of translocation t (1; 12) (q43; q21. 1) in two apparently independent families with vascular phenotypes. Mol. Genet. Genom. Med..

[178] Bouwman A.C., Derks M.F.L., Broekhuijse M.L.W.J., Harlizius B., Veerkamp R.F. (2020). Using short read sequencing to characterise balanced reciprocal translocations in pigs. BMC Genom..

[179] Donaldson B. (2020). Reciprocal Chromosome Translocations in the Domestic Pig, the Prevalence, Genetic and Genomic Factors Associated with Breakpoint Formation. Ph.D. Thesis.

[180] Wetterstrand K.A. (2020). DNA Sequencing Costs: Data from the NHGRI Genome Sequencing Program (GSP). www.genome.gov/sequencingcostsdata.

[181] Madan K., Ford C.E., Polge C. (1978). A reciprocal translocation, t (6p+; 14q−), in the pig. Reproduction.

